# Characteristics of bacteria based self healing rubberized concrete for sustainable and durable construction

**DOI:** 10.1038/s41598-025-97174-1

**Published:** 2025-04-28

**Authors:** Abeer M. Eisa, Ahmed M. Tahwia, Yehia A. Osman, Walid E. Elemam

**Affiliations:** 1 Department of Structural Engineering, Faculty of Engineering, Mansoura University, Mansoura, Egypt; 2 Department of Microbiology, Faculty of Science, Mansoura University, Mansoura, Egypt

**Keywords:** Self-healing concrete, Rubberized concrete, Crack filling, Bacterial concentration, Mechanical performance, Microstructure, Materials science, Civil engineering

## Abstract

Developing longer-lifespan concrete with minimized surface cracking is crucial for sustainable construction. This study investigates self-healing rubberized concrete incorporating 15% recycled rubber waste as a sand replacement. To enhance strength and flexibility through crack closure, bacteria Sporosarcina Pasteurii and Rhizobium Leguminosarum were introduced at 20% of the water volume. Slump, compressive and flexural strength, SEM, and EDX were the tests performed to identify the effects of bacteria and rubber on the concrete characteristics. The results illustrated that the use of rubber as a partial replacement for sand significantly reduced concrete workability and mechanical performance, with slump, compressive strength, and flexural strength decreasing by up to 77%, 49%, and 47%, respectively. However, incorporating SpP and RL bacteria, particularly at concentrations of 10^10^ + 10^10^ and 10^14^ + 10^10^, effectively mitigated these negative effects. The improvement in compressive strength and flexural strength was up to 98.7% and 137.4%, respectively for mixture containing SpP and RL bacteria at concentration 10^10^ +10^10^ compared to mixture containing 15% rubber only. Complete crack self-healing was achieved in SHRC mixtures after 80 days. Microstructure analysis revealed that the formation of calcium carbonate in large quantities within the concrete matrix, which works to heal cracks and fill voids. Thus, using rubber with bacteria to heal cracks could be a cost-effective solution that helps to increase tire rubber recycling rates.

## Introduction

Concrete is a composite building material consisting of aggregate, cement, and water. It is regarded as the building material that is most frequently utilized worldwide^1,2^. Concrete exhibits strong compression strength, weak tension strength, and low crack resistance^3,4^. Concrete cracks can be caused by a variety of factors, such as poor design, irregular settling, environmental variables, plastic shrinkage, and continuous load application. Concrete cracks are practically inevitable^5–7^. Cracks lead to failures and catastrophes in construction structures^8–10^. The permeability of the concrete may be impacted by an ongoing web of microcracks, reducing the material’s ability to withstand the infiltration of toxic materials^11^. Even though they are not immediately considered a threat to the building’s structural integrity, microcracks (up to 0.4 mm) are undesirable according to conventional design regulations^12^. Producing cement-based materials with a better ability to deform before cracking would be the most practical course of action in such situations^13^. Scrap tires are one of the main wastes produced each year as a result of the large amount of vehicles on the road^14–16^. These tires can also be used for civil engineering applications, tire-derived fuel, and ground rubber^17^. Additionally, adding used tires to concrete can improve its flexural strength and other properties^18,19^. Therefore, adding used tires to concrete not only enhances some of its properties but also reduces aggregate usage, saves money, and benefits the environment^20–24^. However, crumb rubber concrete has lower compressive strength^25,26^, elastic modulus^27^, tensile strength^28^, workability^29,30^, and durability performance^31,32^. Li et al.^33^ used the split-Hopkinson pressure bar test to investigate the impact of mechanical properties of waste rubber-modified recycled aggregate concrete and discovered that it exhibits excellent impact conduct. Kashani et al.^34^ found that adding recycled tire crumb to lightweight cellular concrete improved its sound and heat absorption properties. Rubberized concrete (RC) has several advantages over regular concrete, including the efficient use of waste rubbers, the conservation of natural resources, and the building of a path for long-term economic and social development. When compared to the standard mixture, Bing and Ning^35^ found that the slump was decreased by more than 12.7 mm (0.5 in) when 25% of the coarse aggregate was replaced with rubber particles. Higher replacement percentages of rubber significantly decreased the slump. Concrete structure repair is made possible by the multitude of available repair methods^3^. The self-healing method is particularly useful for repairing small and deep cracks in concrete and can also prevent early cracks from developing into large cracks^36^. In recent years, self-healing concrete fractures utilizing a range of bacterial species have been widely used to enhance the properties of concrete and increase its lifespan^37^. It has been observed that the bacteria Sporosarcina pasturii can seal small cracks and cause them to self-heal by filling the cracks with CaCO_3_. This increases the compressive strength of the concrete and improves its resistance to water penetration. When water and dissolved carbon dioxide reach the cracks, the cracks can heal and repair themselves by precipitating calcium carbonate^13,38–40^. The self-healing properties of mortar and concrete were emphasized by Tziviloglou et al.^41^. They demonstrated how bacteria that were cultivated in an alkaline environment thrived considerably better in the surroundings. Furthermore, the trials were designed to achieve the targeted long-term properties with fewer pores in the buildings, and the outcomes demonstrated positive progress toward these goals. Sarkar et al.^42^ showed that the maximum compressive strength was attained at a cell concentration of 10^5^ cells/ml, even for the genetically modified Bacillus subtilis. Additionally, Andalib and colleagues^43^ utilized five distinct Bacillus magaterium cell concentrations (10 × 10^5^–50 × 10^5^ cfu/ml) and discovered that 30 × 10^5^ cfu/ml was the optimal concentration for enhancing strength. Three distinct bacterial concentrations (10^3^, 10^5^, and 10^7^ cells/ml) were used by Chahal et al.^4^ to determine the maximum improvement in compressive strength of fly ash concrete, which was 22% higher at 10^5^ cells/ml of Sporoscarcina pasteurii. By using lightweight aggregate with Bacillus subtilis at a bacterial cell concentration of 3 × 10^8^ cells/cm^3^, Khaliq et al.^44^ observed a 12% increase in compressive strength. Kumari et al.^45^ used three different cell concentrations (10^5^, 10^6^, and 10^7^cfu/ml) of Bacillus conhii and discovered that the bacterial cell concentration of 10^7^ cells/ml resulted in the greatest strength gain of 49.18%. Luo et al.^46^ reported that the bacterial strains that make up Bacillus sphaericus. It led to the formation of calcium carbonate, which is responsible for healing due to urease activity. It was found that 80 days after crack formation, spores forming unknown alkaloid bacteria completely healed large cracks up to a crack width of 0.48 mm. The self-healing technique is confined to small fissures and operates only when water is present^47^. In the concrete industry, self-repair technology is a relatively new term for materials with excellent quality and the capacity to repair damage on their own without the assistance of an outside party^48^.

While previous studies have primarily focused on rubberized concrete’s mechanical characteristics, they repeatedly noted significant drawbacks, such as decreased workability, compressive strength, tensile strength, elastic modulus, and durability in comparison to normal concrete. Limited research has been conducted to explore effective solutions to these challenges, and to investigate the potential role of biological additions in improving rubberized concrete characteristics. This study focused on addressing this research gap by assessing the possibility of introducing two bacterial strains, Sporosarcina pasteurii (SpP) and Rhizobium leguminosarum (RL), into rubberized concrete to improve its workability, mechanical performance, and self-healing capacity. Nine types of self-healing rubberized concrete (SHRC) samples with various concentrations (10^8^, 10^10^ and 10^14^ cell/mL) and a constant ratio of rubber particles of 15% as a replacement of sand and a constant ratio of bacteria percentage of 20% from volume of water. This innovative approach investigates how bacteria and rubber work together to develop more sustainable, longer-lifespan concrete solutions.

## Experimental program

### Concrete constituent materials

**Fig. 1:**
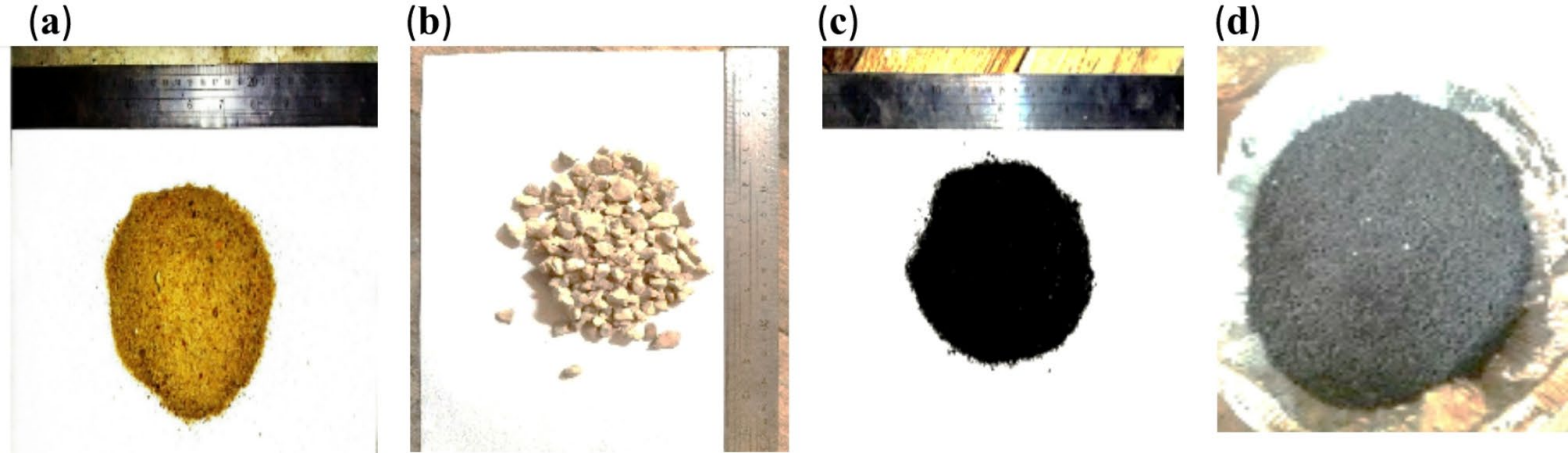
(**a**) Natural sand; (**b**) Crushed dolomite; (**c**) rubber particles size (0.2/2 mm); (**d**) rubber particles size (2/4 mm).

**Fig. 2:**
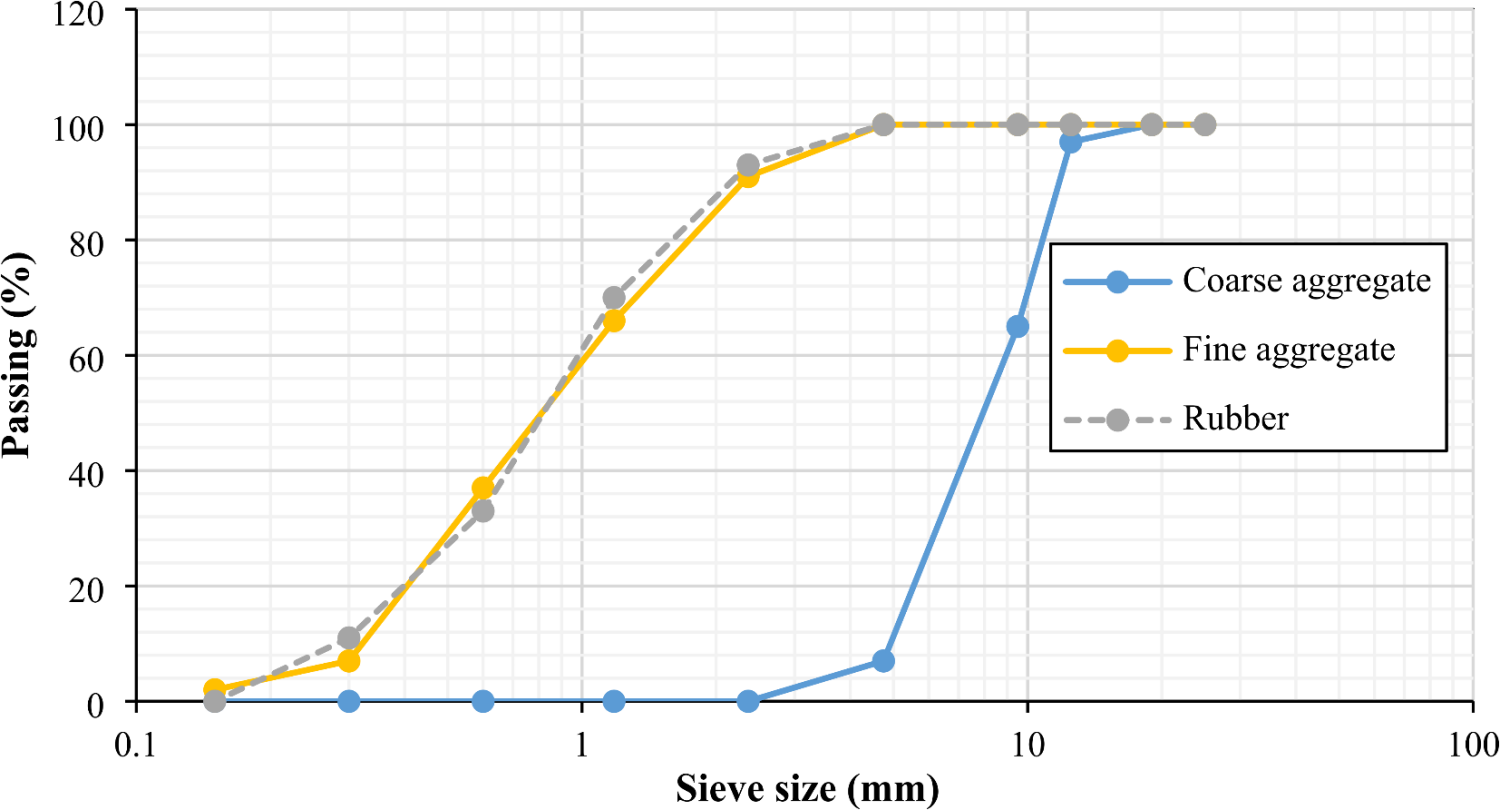
Grain size distributions of coarse aggregate, fine aggregate, and rubber particles.

In this study, ordinary Portland cement (OPC) type CEM I 42.5 N, which complies with BS EN 197-1/2011^49^ was used. Coarse aggregate (crushed dolomite) with 4/12 mm size, and locally available natural fine aggregate (river sand) with 0/4 mm size and 2.95 fineness modulus were utilized in accordance with BS EN 12,620^50^. Figure 1a and b shows the fine and coarse aggregate used in this investigation. In the mixing process, pure drinking water was used to maintain the integrity of the concrete, ensuring consistent strength development and preventing any negative effects on the appearance and surface quality of the hardened concrete. The water-to-cement ratio (w/c) was 0.45. Two bacterial strains: Sporosarcina Pasturii (SpP) (bio cement-producing bacteria) and Rhizobium leguminosarum (RL) (biofilm product) were used at concentrations of 10^8^, 10^10^ and 10^14^. Two sizes of crumb rubber (0.2–2 mm and 2–4 mm), as depicted in Fig. 1c and d, were used as a partial replacement for sand at 15% by volume. To maintain a grading curve almost similar to that of natural sand, the fine rubber particles (2–4 mm) were blended with smaller-sized particles (0.2–2 mm) in a 1:1 ratio. The crumb rubber had a specific gravity of 0.98, and bulk density of 0.55 t/m^3^. Figure 2 shows the grain size distribution of all aggregates used in this investigation. Additionally, a superplasticizer (SP) conforming to ASTM C494 Type F^51^ was used at a fixed dosage of 3% by weight of cement to reduce the water-cement ratio and enhance workability, thereby improving both mechanical properties and durability.

### Preparation of bacterial self-healing factor

**Fig. 3:**
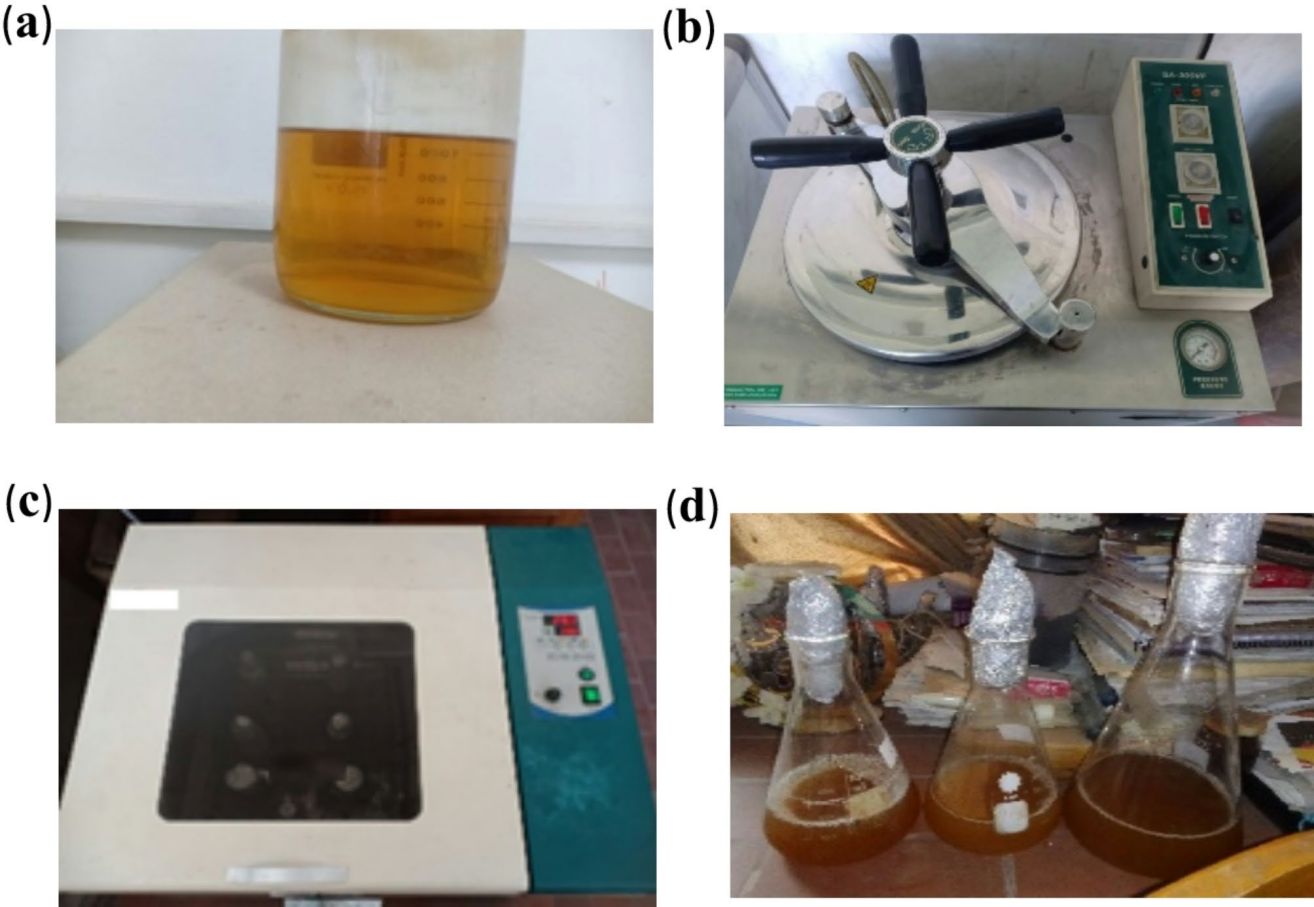
Process of obtaining bacterial spores. (**a**) collect nutrient broth medium; (**b**) incubation using an autoclave; (**c**) orbital shaker; (**d**) final form of bacteria in flasks.

Bacteria (SpP and RL) were collected and verified for cultivation suitability. A sterile liquid medium (Nutrient Broth) was prepared by dissolving 40 gm of peptic digest of animal tissue and 6 gm of yeast extract per liter of water (pH = 7.2). The mixture was autoclaved at 121 °C for 25 min to ensure sterility. The sterile medium was inoculated with bacterial strains and incubated at 30 °C on a shaker set at 170 rpm for 48 h. This horizontal circular shaking motion ensured uniform mixing and aeration for bacterial growth. After incubation, bacterial cells were separated by centrifuging the culture medium in flasks. This process yielded the concentrated bacterial cells for use in concrete mixtures, as illustrated in Fig. 3.

### Preparing of concrete samples

As indicated in Table 1, a total of eleven different concrete mixtures were designed for this study. Concrete mixtures were prepared in which part of the sand was replaced by rubber particles to produce less brittle concrete. Bacteria constitute 20% of the total water mass at all concentrations of 10^8^, 10^10^, and 10^14^. The rubber, cement, coarse aggregates (CA), and fine aggregates (FA) were first mixed in the mixer for 3 min, then water and bacteria were added to the dry ingredients. Finally, mix all the ingredients well to obtain the concrete mixture for 5 min.

**Table 1:** Mixing proportions of the concrete specimens.

Mix	Cement (kg/m^3^)	CA (kg/m^3^)	FA (kg/m^3^)	Rubber (kg/m^3^)		Bacteria (10%SpP + 10%RL) (cell/m^3^)	Water (kg/m^3^)	Super plasticizer (kg/m^3^)
				0.2/2 mm	2/4 mm			
**M0**	375	1096	730	–	–	–	170	11.25
**M1**	375	1096	620.5	21	21	–	170	11.25
**M2**	375	1096	620.5	21	21	10^8^ +10^8^	170	11.25
**M3**	375	1096	620.5	21	21	10^10^ +10^8^	170	11.25
**M4**	375	1096	620.5	21	21	10^14^ +10^8^	170	11.25
**M5**	375	1096	620.5	21	21	10^8^ +10^10^	170	11.25
**M6**	375	1096	620.5	21	21	10^10^ +10^10^	170	11.25
**M7**	375	1096	620.5	21	21	10^14^ +10^10^	170	11.25
**M8**	375	1096	620.5	21	21	10^8^ +10^14^	170	11.25
**M9**	375	1096	620.5	21	21	10^10^ +10^14^	170	11.25
**M10**	375	1096	620.5	21	21	10^14^ +10^14^	170	11.25

### Testing of fresh concrete

The workability of concrete affects its density and hardened properties. The slump test was performed according to ASTM C143^52^ to determine the impact of rubber particles, and bacteria (SpP and RL) on concrete workability.

### Testing of hardened concrete

#### Compressive strength

The compressive strength of the composite specimens was determined according to BS EN 12390-3^53^. Nine cubes with a size of 100 × 100 × 100 mm were used to determine compressive strength at age 7, 28 and 56 days (three samples for each period).

#### Flexural strength

Flexural testing is generally used to determine the flexural modulus or flexural strength of a particular material^54^. Studying flexural strength on concrete is important to maintain its durability by preventing cracking and corrosion. Design considerations must be taken into account^55^. The procedure outlined in the ASTM-C78-16^56^ standard was employed to ascertain the flexural strength. Tests were performed on samples measuring 100 × 100 × 500 mm at 28 days, using the three-point method.

#### Microstructure

Energy Dispersive Spectroscopy (EDX) and Scanning Electron Microscopy (SEM) are advanced techniques used for phase determination, chemical characterization of unknown elements, and identification of specific defects in concrete samples, such as voids, micro-cracks, and chemical inconsistencies commonly found in water-based cement paste for concrete^57,58^. In order to get specimens for SEM imaging, concrete samples were taken from the center of the concrete cube. While the EDX test was performed on a sample of concrete after grinding. At 56 days of age, SEM and EDX testing were conducted. In this study, four mixtures (M0, M1, M2 and M6) were examined.

### Creation of cracks

**Fig. 4:**
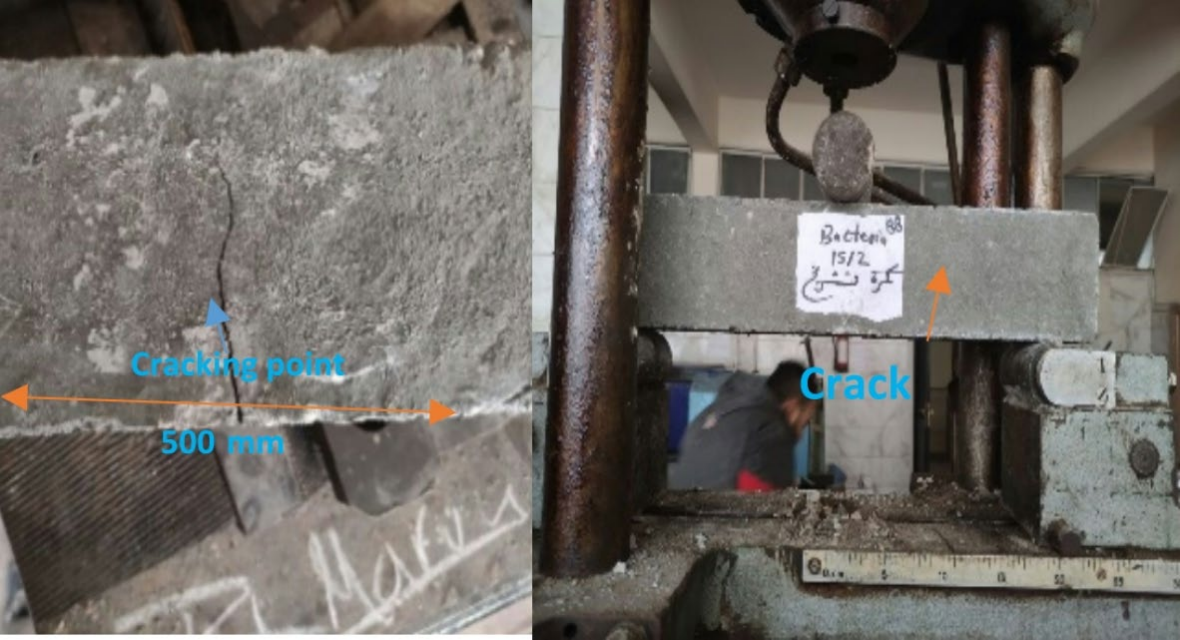
The creation of cracks.

In this research, cracks were created in three concrete samples (100 × 100 × 500) mm for each mix to monitor the ability of each type of concrete to self-heal. Concrete was subject to water curing for 7- days, in accordance with BS EN 12390-3:2019^59^, and then specific specimens were subjected to 30–40% of the ultimate load to create micro-cracks with different widths in the samples, as shown in Fig. 4. Three samples of each concrete mixture were loaded with different loads to develop cracks with varying widths ranging from 0.41 to 1.25 mm, after which the samples were water cured again.

## Results and discussion

### Slump

**Fig. 5:**
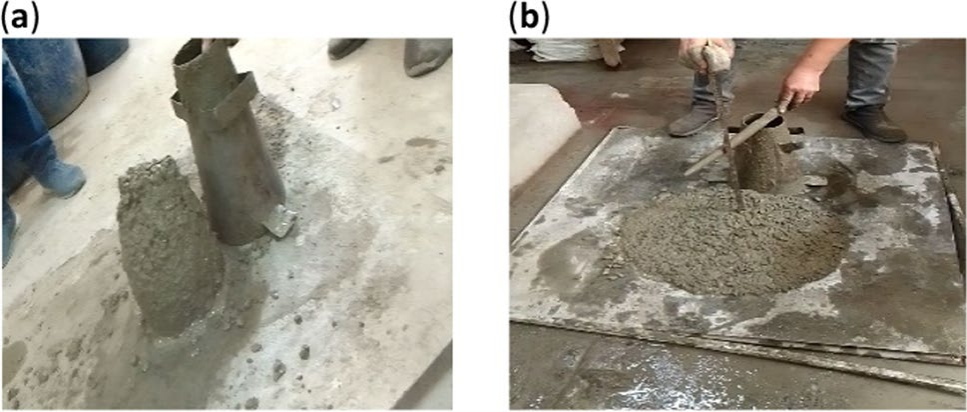
Result of the slump test of (**a**) rubberized concrete, and (**b**) SHRC (M10).

**Fig. 6:**
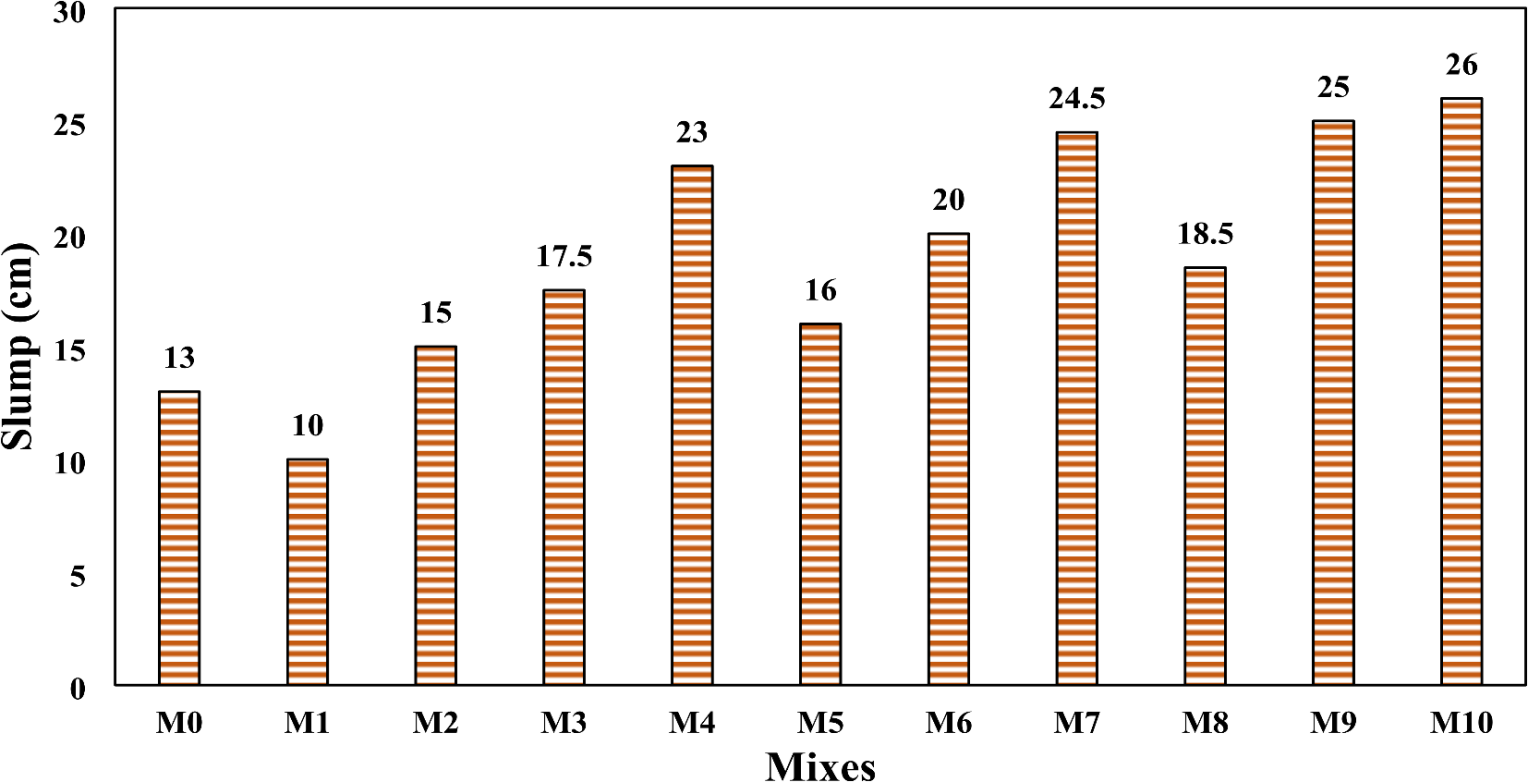
Slump values of all concrete samples.

Figure 5 illustrates the slump values for all the designed concrete mixtures. The slump value for the control mix (M0) reached 13 cm. However, upon incorporating rubber particles into the mix (M1), a significant reduction in slump value was observed, indicating a notable decrease in workability. This decline can be attributed to the irregular shape and surface texture of rubber particles, which hindered the flow of the concrete mix. These findings are consistent with the observations reported by Khatib and Bayoumi^60^ and Abbas et al.^61^. This is due to the ability of rubber particles to absorb free water from the fresh concrete into the voids on their surface, reducing the flow of the fresh mix. The addition of bacteria to the concrete had a good influence on the slump findings, as illustrated in Fig. 6. The percentage of increase in slump value up to 100% for mix M10 which contained SpP and RL bacteria with concentration 10^14^ + 10^14^ followed by mix M9 which containing bacteria with concentration 10^10^ +10^14^ compared to control mix and up to 160% and 150%, respectively compared to M1. The results clearly indicate that as the concentration of RL and SpP bacteria increases, the slump value rises, enhancing the workability of the concrete. The slump value reached 20 cm for mix M6, which contained SpP and RL bacteria at a concentration of 10^10^ + 10^10^, compared to 15 cm for mix M2, which contained bacteria at a concentration of 10^8^ + 10^8^. Notably, when the bacterial concentration increased to 10¹⁴ + 10¹⁴ in mix M10, the slump value further increased to 26 cm, demonstrating a positive correlation between bacterial concentration and workability. These results are agreed with Xu et al.^62^, and Ding and Ning^35^. These findings indicate that a more fluidity mix was produced by the healing agent’s presence. However, contrasting results by Kashif et al.^63^ indicated a slight reduction in fluidity with the introduction of bacterial cultures, possibly due to the influence of bacterial spores on the mix’s consistency.

### Compressive strength

Table 2 shows the results of compressive strength for all mixtures at 7, 28, and 56 days. It was observed that variations in the mixture components and bacteria concentration led to a significant difference in the concrete’s compressive strength. The incorporation of bacteria, along with changes in the mix composition, had a notable impact on improving the strength, particularly at higher bacterial concentrations. The comparison between mix M0 and mix M1 reveals that the partial replacement of sand with rubber had a significant negative impact on compressive strength. This reduction is attributed to the inherent properties of rubber particles, which create weak zones within the concrete matrix, compromising its load-bearing capacity. These findings align with the observations of Khatib and Bayoumi^60^, who reported a similar decline in compressive strength due to the inclusion of rubber particles. In contrast, SHRC mixes that included SpP and RL bacteria as a partial substitute for water showed a significant increase in compressive strength when compared to RC mix. The primary cause of this improvement is the SpP bacteria’s ability to produce CaCO_3_. The overall strength of concrete and structural integrity are enhanced by the precipitated CaCO₃, which efficiently fills in voids and microcracks.0.

**Fig. 7:**
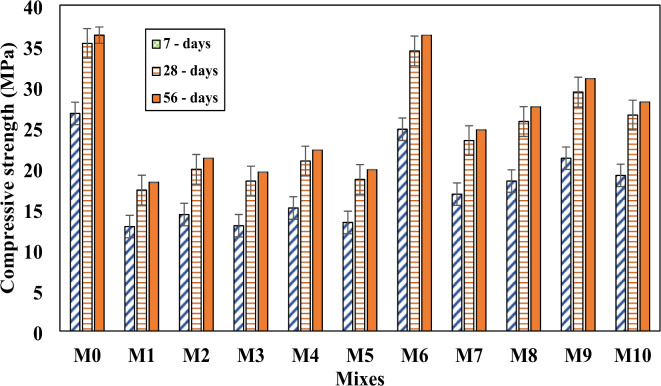
Compressive strength for all mixes after 7, 28, and 56 days.

This reduced pore size and increased compressive strength by filling cracks and voids. On the other hand, RL used in concrete has been shown to act as a partial glue to bind particles together and fill pores to reduce the size of holes and enhance strength^64^. The highest compressive strength was achieved at concentrations of 10^10^ + 10^10^ followed by 10^10^ + 10^14^ as shown in Fig. 7. The specific concentration of 10^10^+10^10^ of bacteria exhibited the highest improvement in concrete strength may be due to the optimal balance between bacterial activity and the availability of nutrients for their growth. The percentage of increase in compressive strength was 98.7% and 69.5%, respectively compared to the RC mix, at age 28 day. This percentage of increase in compressive strength was also achieved at age 56 days. The results clearly demonstrated a significant impact of incorporating bacteria in mitigating the negative effects of rubber on the compressive strength of concrete. Specifically, mix M6, which contained SpP and RL bacteria, achieved a compressive strength nearly equivalent to that of the control mix. The compressive strength of mix M6 achieved 92.8%, 97.4%, and 99.94% of control mix strength. The compressive strength of mix M6 reached 92.8%, 97.4%, and 99.94% of the control mix strength at 7, 28, and 56 days, respectively. The progressive improvement suggests that the bacteria’s self-healing action continues to enhance the concrete’s performance throughout the curing period. This suggests that the bacterial activity effectively countered the detrimental influence of rubber particles, restoring and even enhancing the concrete’s strength through mechanisms such as crack healing and CaCO₃ precipitation. The findings agree with those of Algaifi et al.^65^. They demonstrated that, in comparison to regular concrete specimens, the incorporation of microbial calcium carbonate into the self-repairing concrete matrix may improve the compressive strength. Similar findings were reported by Shaheen et al.^66^, who demonstrated that the employment of immobilization techniques for the self-healing process increased the mechanical properties of the generated concrete, such as its compressive strength.

### Flexural strength

**Fig. 8:**
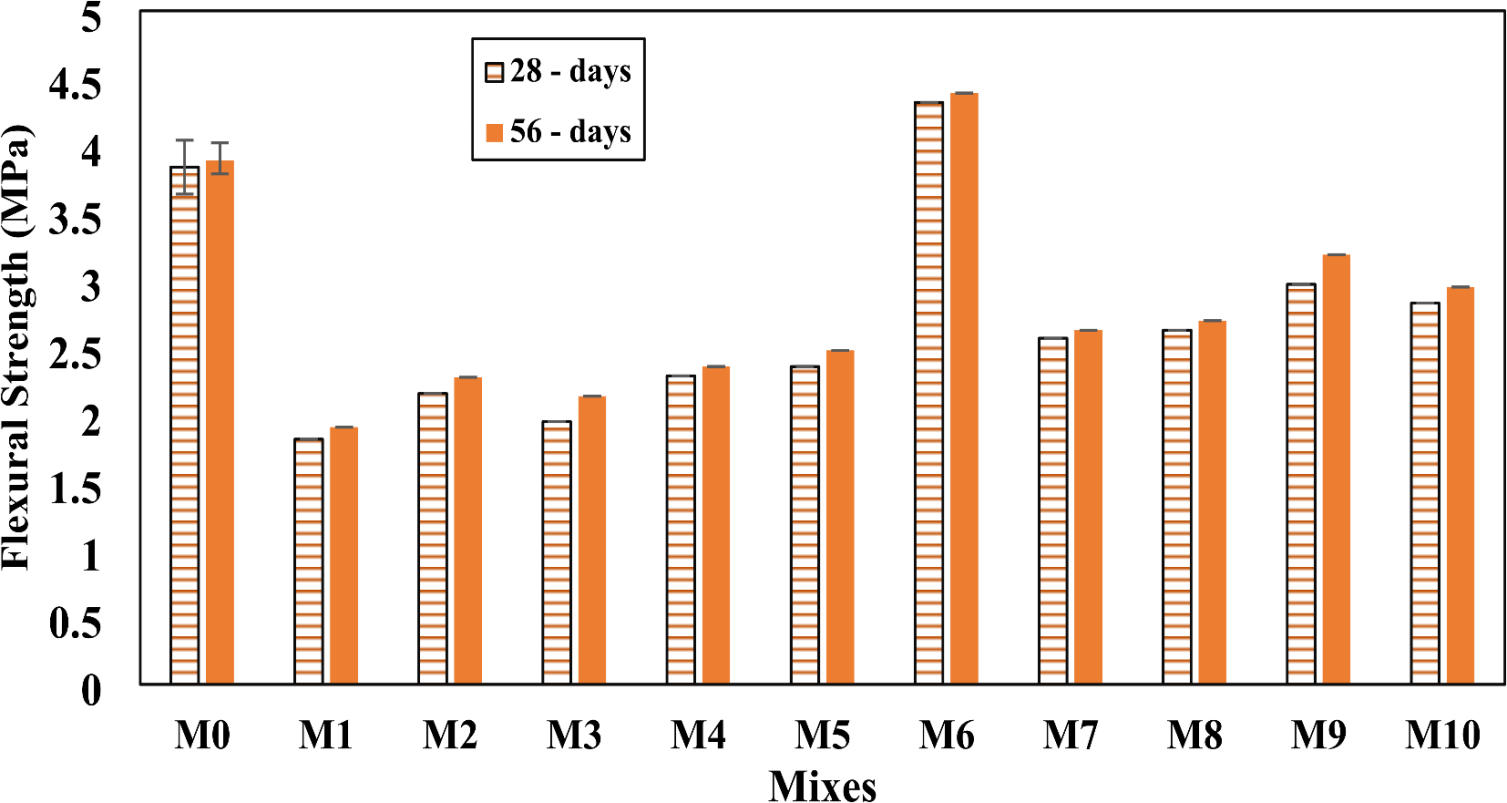
Flexural strength for all mixes after 28 and 56 days.

This feature protects an unreinforced concrete slab or beam against bending failure. The flexural strength of concrete mixes is affected by the type and concentration of bacteria as well as the presence of crumb rubber, as shown in Fig. 8. It is evident that the trends in the results for flexural and compressive strength are similar. Flexural strength results show that a substantial reduction in flexural strength occurred when 15% rubber was used in place of sand. The flexural strength reduced from 3.84 MPa and 3.89 MPa for control mix without rubber to 1.82 MPa and 1.91 MPa for RC mix, at age 28 and 56 days, respectively. The reduction in flexural strength due to the use of crumb rubber can be attributed to that crumb rubber being a soft and elastic material, does not bond well with the cement matrix. This poor bonding results in reduced cohesion between the rubber particles and the surrounding concrete, leading to a weaker flexural strength. This reduction in flexural strength was decreased with the use of bacteria, there was a positive improvement in concrete flexural strength of the mixtures containing bacteria where it exceeded that of the control mix. The use of 20% from bacteria (SpP + RL) enhanced the flexural strength, especially at concentration 10^10^ + 10^10^, with a percentage of increase up to 137.4% compared to the RC mix, and up to 12.5% compared to the control mix, at age 28 days. This percentage of increase in flexural strength was also achieved at age 56 days, as shown in Table 2. This increase in concrete strength may be attributed to that bacteria such as SpP and RL facilitate the production of CaCO₃, which acts as a healing agent in the concrete. This precipitation helps to fill microcracks and voids created by the rubber particles, enhancing the concrete’s density, cohesion, and overall strength. The bacteria essentially restore the structural integrity of the concrete by improving the bonding between particles. These results agree with Metwally et al.^54^, and El-Mahdy and Tahwia^64^.

**Table 2:** Results of the compressive strength and flexural strength tests for all mixes.

Mix	Concentration of bacteria SP×RL (cell/m^3^)	Rubber (kg/m^3^)		Compressive strength (MPa)			Flexural strength (MPa)	
		2/4 mm	0.2/2 mm	7 days	28 days	56 days	28 days	56 days
**M0**	–	–	–	26.7	35.30	36.31	3.84	3.89
**M1**	–	21	21	12.82	17.30	18.25	1.82	1.91
**M2**	10^8^ +10^8^	21	21	14.30	19.85	21.2	2.16	2.28
**M3**	10^10^ +10^8^	21	21	12.93	18.40	19.50	1.95	2.14
**M4**	10^14^ +10^8^	21	21	15.10	20.86	22.20	2.29	2.36
**M5**	10^8^ +10^10^	21	21	14.95	21.40	22.80	2.36	2.48
**M6**	10^10^ +10^10^	21	21	24.77	34.38	36.29	4.32	4.39
**M7**	10^14^ +10^10^	21	21	16.80	23.38	24.68	2.57	2.63
**M8**	10^8^ +10^14^	21	21	18.40	25.71	27.51	2.63	2.70
**M9**	10^10^ +10^14^	21	21	21.20	29.33	30.98	2.97	3.19
**M10**	10^14^ +10^14^	21	21	19.10	26.50	28.10	2.83	2.95

### Concrete microstructure

#### SEM

**Fig. 9:**
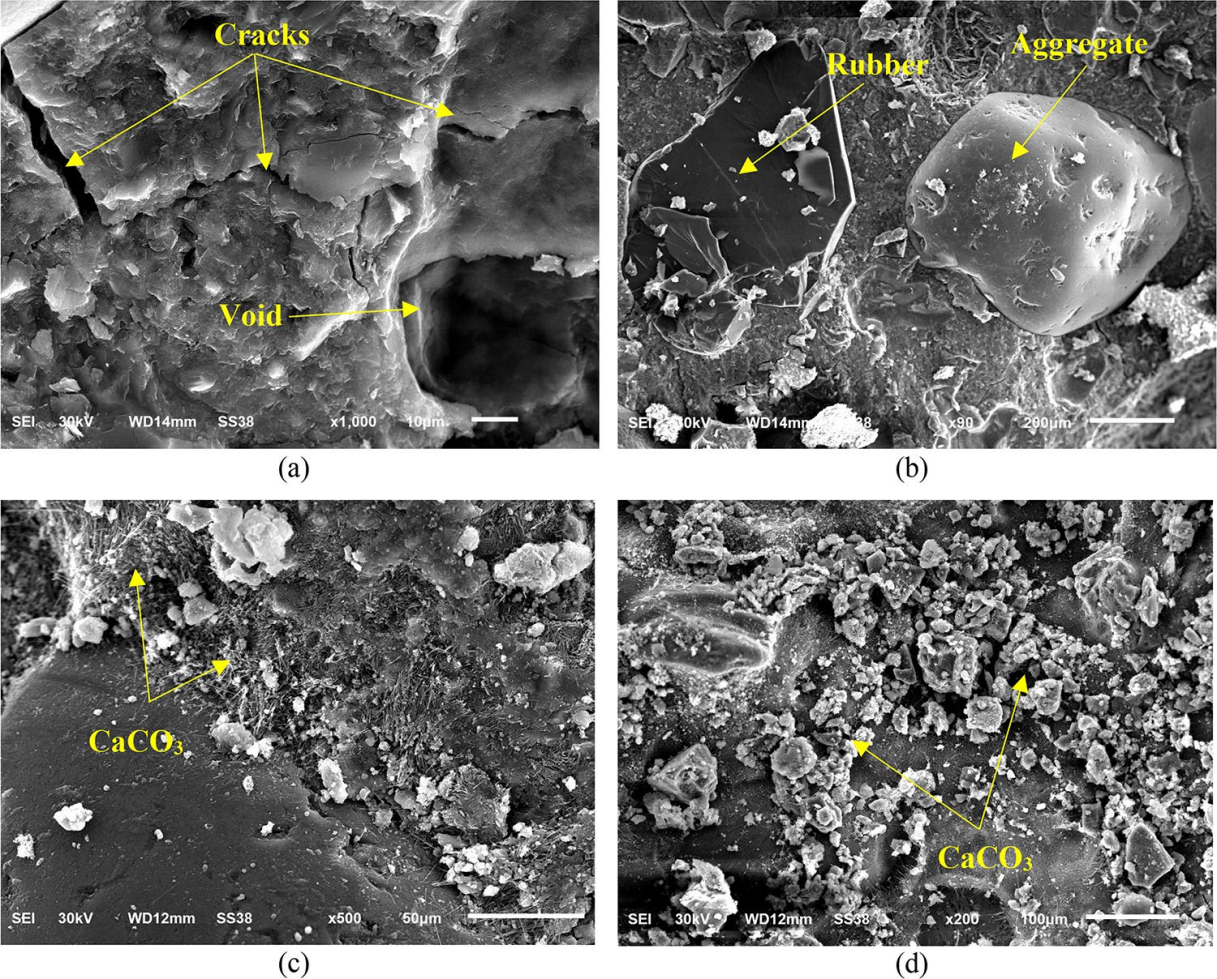
SEM results (1000x) after 56 days of curing for mixes (**a**) control mix (M0); (**b**) M1; (**c**) M2 and (**d**) M6.

SEM was conducted for M1, M2 and M6 mixtures compared to M0. Figure 9 shows the SEM results of the selected mixes after age 56 days at a magnification level of 1000 x. The control mix (M0) exhibited a microstructure with numerous voids, as shown in Fig. 9a, which negatively affected its mechanical properties. However, the introduction of 15% rubber particles as a partial replacement for sand in mix M1, as illustrated in Fig. 9b, resulted in a mixture with fewer voids compared to the control mix. This modification led to an improvement in the distribution of the cementitious matrix, promoting better bonding between particles. While the addition of rubber reduced the compressive and flexural strength relative to the control mix, it improved the overall microstructure by reducing the porosity and enhancing the inter-particle connectivity. The reduced void content contributed to better consolidation, thereby improving the mechanical properties and durability of the concrete, despite the reduction in strength caused by the rubber’s presence. The rubber particles likely acted as fillers that, while reducing strength, also led to a more evenly distributed and dense microstructure, which can contribute to improved durability and long-term performance of the material. On the other hand, a mixture containing 15% rubber as a replacement of sand and 20% bacteria at concentration 10^8^ + 10^8^ contained a smaller amount of CaCO_3_ which has a better dense surface with fewer voids, which led to a slight improvement in the value of compressive and flexural strength compared to M1 (Fig. 9c). Moreover, Fig. 9d shows that the use of 15% rubber as a replacement of sand and 20% bacteria at concentration 10^10^ +10^10^ contained a larger amount CaCO_3_ that exhibited a dense surface with low permeability and no voids, which led to very enhanced in compressive and flexural strength compared with the three mixes another. The densely packed microstructure contributes to lower permeability, as the calcite formed effectively voids and cracks, creating a tighter bond with the cementitious materials. This improved matrix not only enhances the resistance to water and chemical ingress but also strengthens the interfacial transition zone, leading to better mechanical properties, increased durability, and prolonged service life of the concrete. The results revealed a strong relationship between the precipitated calcite and the other concrete components utilized throughout the crack healing process, as seen by the lower void count. Higher density and fewer voids lead to less permeability. Also, the findings illustrate the benefits of incorporating rubber particles and bacteria: the rubber particles enhance the mechanical flexibility of the matrix, while bacterial activity accelerates the deposition of calcite and Rhizobium leguminosarum bacteria act as a partial glue, binding the concrete particles with rubber which leads to further improving the structural density and concrete resistance. These findings are consistent with those of Shah and Huseien^48^, Bakr et al.^67^, and Mahmoud et al.^68^ found that the bio-concrete’s permeability decreased as a result of the increased durability caused by calcite precipitation. Additionally, the values for compressive strength and density were higher.

#### EDX

**Fig. 10:**
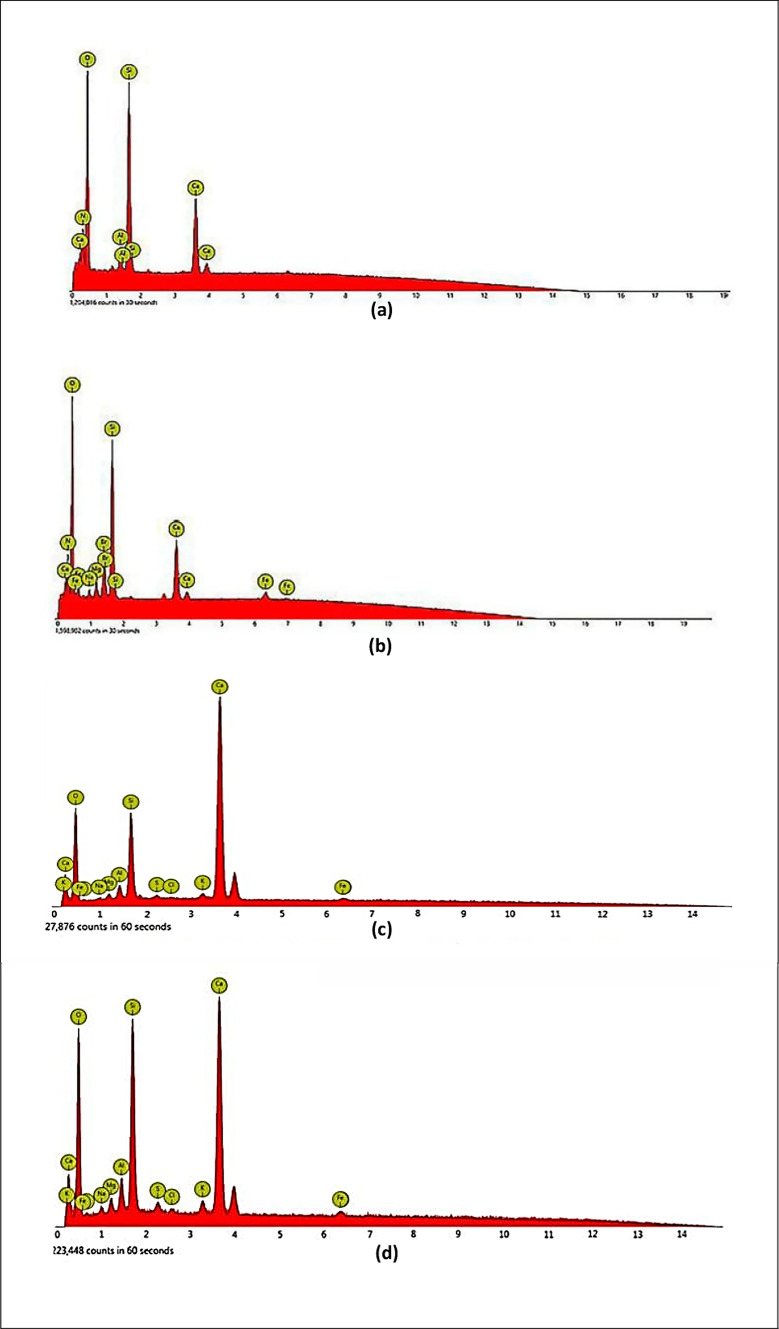
EDX analysis of (**a**) mix M0; (**b**) mix M1; (**c**) mix M2; and (**d**) mix M6.

The element’s peak location will essentially be determined with the use of EDX analysis. Each element’s strength is correlated with how much of it there is in the material. As the dominant element in concrete, oxygen (O) is more common in the control mixture than any other element. Its mass and atomic percentage both decrease as the amount of rubber particles increases in the concrete components. Figure 10 shows the EDX results of the mixes M0, M1, M2, and M6 at age 56 days. Table 3 displayed the element structure of all the concrete specimens obtained from the x-ray micro analysis. The EDX analysis shows significant differences in the elemental composition across the various mixes, particularly in the key elements of calcium (Ca), carbon (C), and sulfur (S). In the control mix M0 (Fig. 10a), calcium comprises 16.20% of the elemental composition while carbon and sulfur comprise 11.6% and 1.2%, respectively. In mix M1 (Fig. 10b), which contains 15% rubber without bacteria, the sulfur and carbon levels rise to 1.80% and 28.17%, respectively, indicating that the rubber particles contribute significantly to the sulfur and carbon content, as tire rubber is known for its high carbon and sulfur content. In mix M2 (Fig. 10c), which contains both rubber and bacteria (SpP and RL) at a concentration of (10^8^ + 10^8^), the calcium percentage increases to 29.96%, indicating enhanced CaCO₃ formation due to the bacterial activity. The bacteria facilitate the precipitation of calcium carbonate, which fills cracks and voids, thereby improving the mechanical properties, durability, and the overall microstructure of the concrete. Similarly, the sulfur content in M2 increases to 1.83%, further supporting the contribution of rubber particles. In mix M6 (Fig. 10d), which contains rubber and bacteria at a higher concentration of 10^10^ + 10^10^, the calcium content rises to 30.06%, reflecting an even higher degree of bacterial activity and more substantial CaCO₃ formation. The increased bacterial concentration appears to further enhance the self-healing capabilities of the concrete. The presence of bacteria at this higher concentration results in a more pronounced improvement in the mechanical properties of concrete due to reduced porosity and more efficient crack repair. The analysis also shows that the aluminum (Al) content decreases slightly from 2.40% in M0 to 2.35% in M1, and increased to 2.89% and 2.94% in M2 and M6, respectively. This suggests that the inclusion of rubber may affect the aluminum content, potentially due to interactions with other materials in the mix. These results are consistent with Akinyele et al.^69^; it is assumed that the relatively high amounts of these two elements come from the rubber particles because tire rubber has very high amounts of sulfur and carbon black. The calcium, oxygen, and carbon atoms that were the main components of the formed powder are confirmed by the EDX analysis of the microbially created calcium carbonate. The particles may have been made by immobilized bacteria because the component compositions of the bio-precipitates resembled those of transparent calcium carbonate crystals. These results agree with Seifan et al.^70^.

**Table 3:** EDX quantification of mixes.

Mix	C	O	Ca	Si	K	Al	S	Fe	Na	Mg
**M0**	11.60	39.21	16.20	26.09	0.7	2.40	1.20	2.60	–	–
**M1**	12.92	28.17	21.77	27.60	0.7	2.35	1.80	2.51	0.69	1.49
**M2**	13.90	19.67	29.96	27.04	0.7	2.89	1.83	1.74	0.73	1.54
**M6**	14.02	19.80	30.06	26.40	0.7	2.94	1.90	1.80	0.78	1.60

### Optical determination of crack-healing capacity

**Fig. 11:**
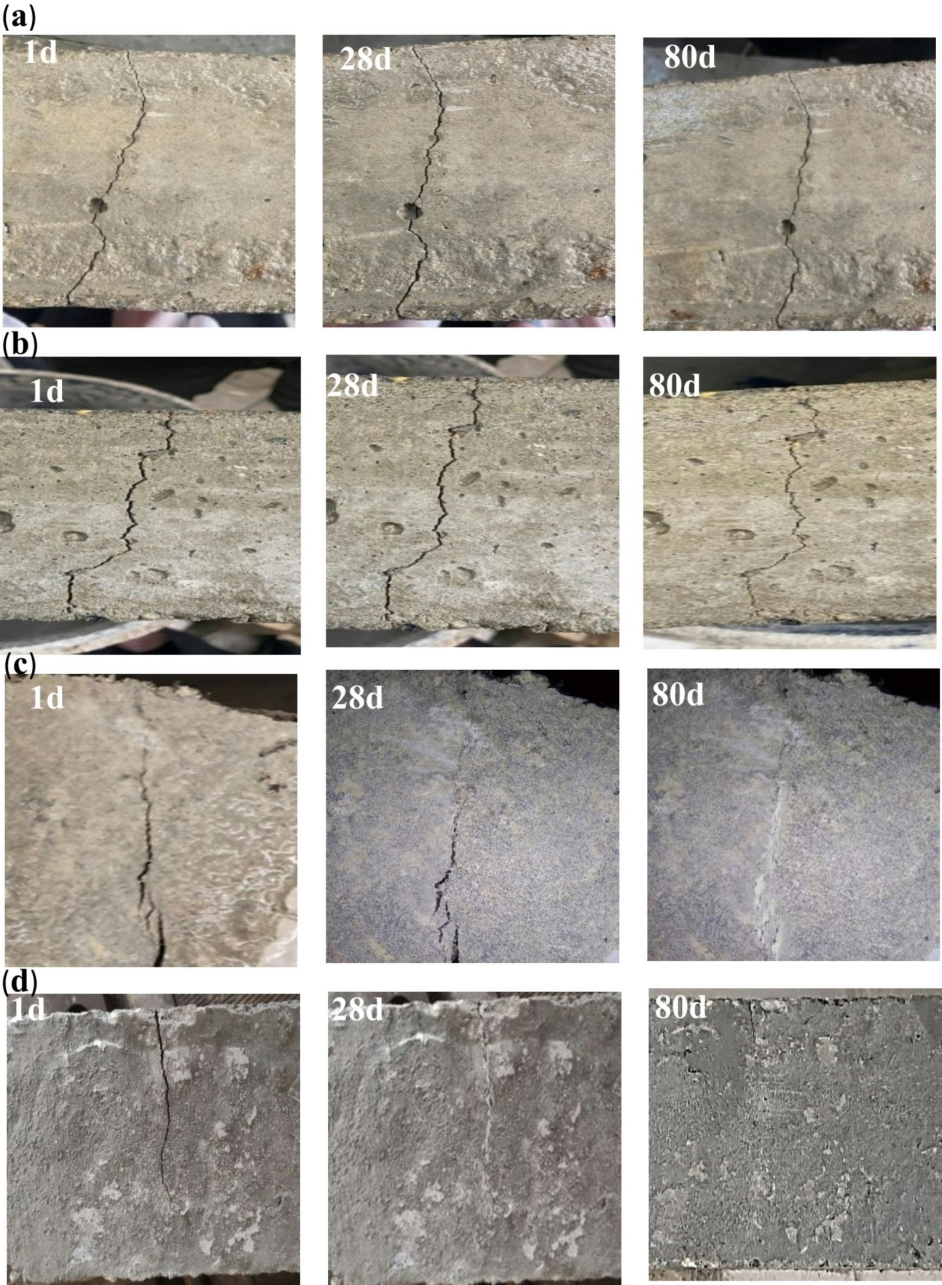
Healing of cracks at different ages for (**a**) control mix M0; (**b**) mix M1; (**c**) mix M6 and (**d**) mix M9.

When creating cracks in SHRC specimens, Vernier calipers were used to measure the various crack widths and depths of the specimens, which ranged from 0.41 to 1.25 mm. Figure 11 shows different pictures of representative healing of cracks at different ages for mixes M0, M1, M6 and M9. It was clearly observed that the cracks in the control mix and RC are not healing compared with SHRC which was repaired and the voids in it have been filled. After 28 days of healing, white precipitates partially covered the surface of the samples, some voids were filled by the CaCO_3_, which means that the concrete’s resistance and durability are still weak. After 80 days, the cracks in SHRC mixtures were completely healed which means that the concrete’s resistance has improved significantly as shown in (Fig. 11c and d), the percentage of healing in M6 after 28 days 90%, and 100% after 80 days and the percentage of healing in M9 after 28 days 88%, and 100% after 80 days compared to mixes M0 (Fig. 11a) and M1(Fig. 11b). The bacteria’s biological activity accelerates the production of CaCO_3_, which fills cracks and strengthens the interfacial zones, improving the repaired concrete’s mechanical performance and durability^37^. These results agreed with Baker et al.^7^, Tziviloglou et al.^71^, and Amran et al.^47^. According to the findings, the self-healing technique that combines rubber particles with two distinct kinds of bacteria is successful in improving concrete’s ability for self-healing.

**Fig. 12:**
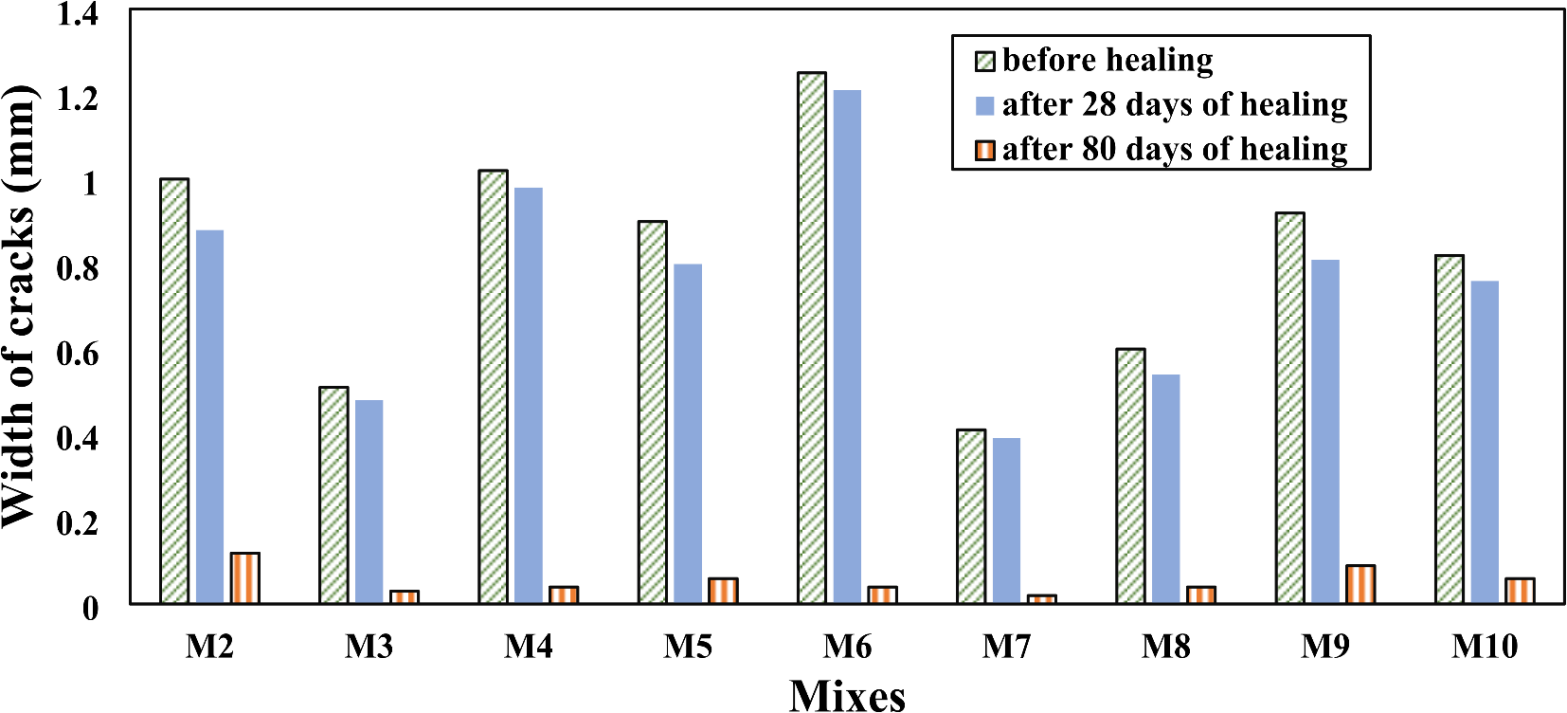
Cracks width of SHRC samples before and after healing.

**Fig. 13:**
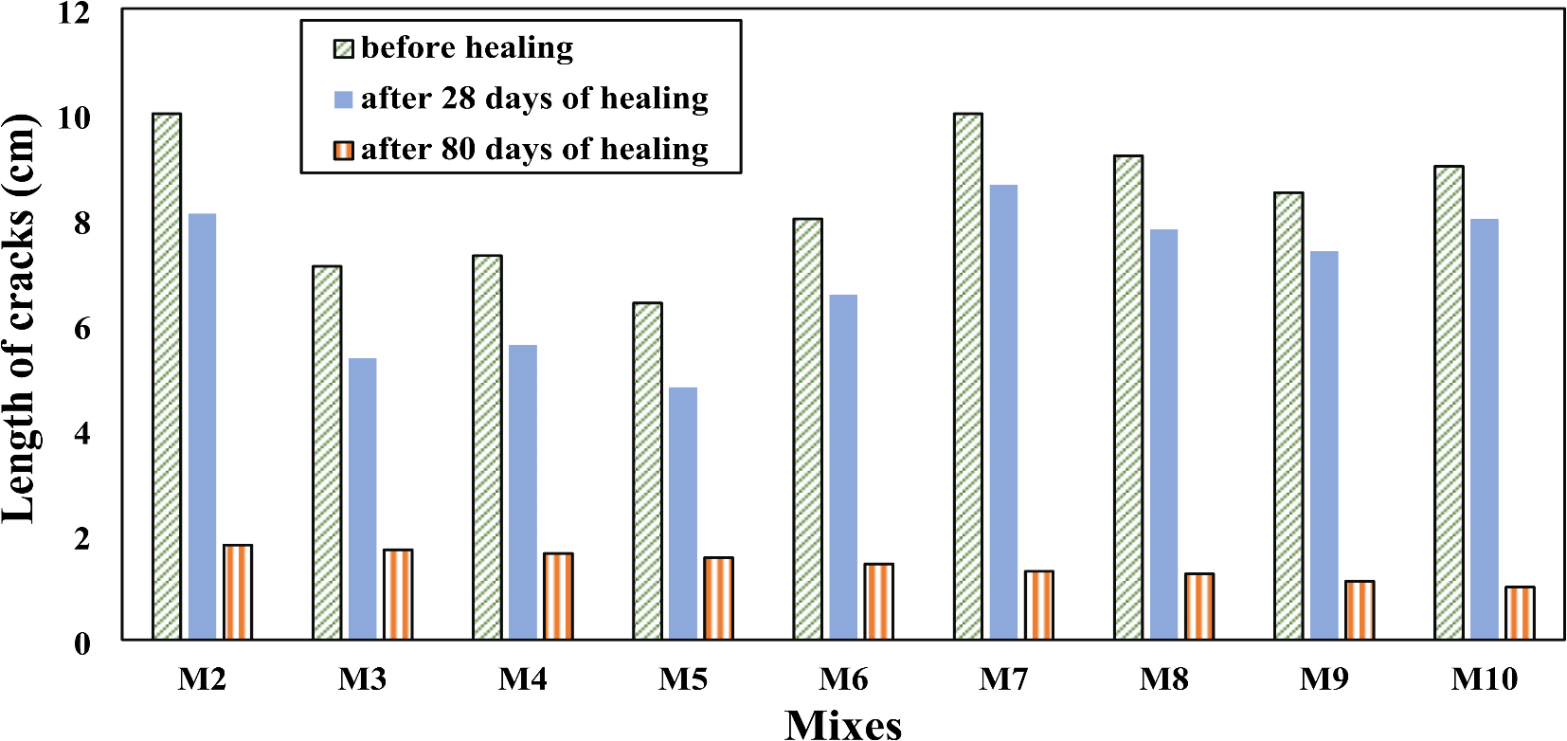
Cracks length of SHRC samples before and after healing.

**Fig. 14:**
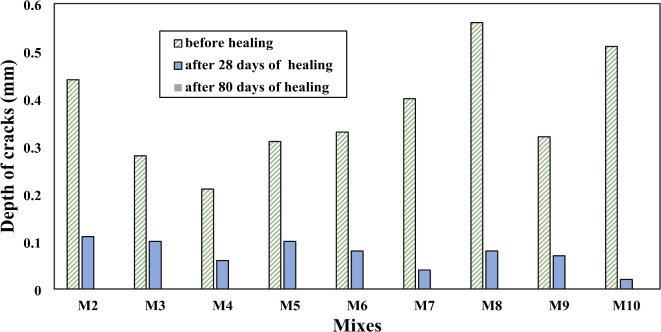
Cracks Depth of SHRC samples before and after healing.

Figure 12 shows the values of fully healed cracks for SHRC at different healing times. With increasing healing time, SHRC samples showed a clear improvement in repairing cracks in concrete, especially concrete samples that contain a high concentration of SpP bacteria, which are responsible for producing CaCO_3_, which in turn serves to heal cracks. It is observed that more than 90% of crack width was healed after 28 days and approximately completely healed after 80 days. Comparing the length of the cracks before healing and after 28 days and 80 days of healing for SHRC mixtures, it observed that more than 80% of crack length was healed after 28 days and approximately completely healed after 80 days as shown in (Fig. 13). Figure 14 shows the depth of cracks in SHRC mixtures before and after healing at different healing periods. Approximately 90% of the crack depth was filled with CaCO_3_ at 28 days and completely filled after 80 days. These results are consistent with those of Luo et al.^46^ with this adaptation found that 80 days after the appearance of cracks, spores forming unknown alkali-loving bacteria completely healed large cracks up to a crack width of 0.48 mm.

## Conclusion

This study examined the possibility of introducing two bacterial strains, Sporosarcina pasteurii (SpP) and Rhizobium leguminosarum (RL), into rubberized concrete to improve its workability, mechanical performance, and self-healing capacity. The research findings can be summarized as follows:

1. The partial replacement of sand with rubber significantly decreased the workability and mechanical characteristic of concrete, the reduction in slump, compressive strength, and flexural strength up to 77%, 49% and 47%, respectively.
2. The addition of SpP and RL bacteria significantly improved the slump value of rubberized concrete, with the highest increase in fluidity observed in mixtures containing higher bacterial concentrations (10^14^ + 10^14^).
3. The incorporation of SpP and RL bacteria in SHRC mitigates the negative impact of rubber particles on compressive strength by reducing pore size and filling cracks with CaCO₃, achieving up to a 98.7% increase in compressive strength at bacterial concentrations of 10¹⁰ + 10¹⁰ compared to rubberized concrete without bacteria. This enhancement brings the performance of SHRC close to that of control mixe, demonstrating the potential of bacterial additives to restore and enhance the mechanical properties of rubberized concrete.
4. The incorporation of SpP and RL bacteria significantly improved the flexural strength of rubberized concrete, with a notable increase of up to 137.4% compared to the rubberized concrete mix, and 12.5% compared to the control mix at 28 days.
5. The microstructure analyses showed that adding SpP and RL bacteria to rubberized concrete reduced voids, increased CaCO₃ precipitation, and improved microstructure, density, and permeability. Bacteria-enhanced mixes also had the highest calcium levels, confirming microbial CaCO₃ formation and microstructure enhancement.
6. The incorporation of SpP and RL bacteria in rubberized concrete significantly enhanced its crack-healing capacity, with over 80% of cracks healed after 28 days and nearly complete healing achieved after 80 days. The formation of CaCO₃ through bacterial activity filled voids and repaired cracks, confirming the potential of SHRC for self-repair and improved mechanical performance over time. This self-healing mechanism proves to be an effective way to extend the service life of concrete structures, especially in applications where crack formation is inevitable.

## Data Availability

The required data has been presented and made available within the manuscript.
